# Conservative Treatment in Avascular Necrosis of the Femoral Head: A Systematic Review

**DOI:** 10.3390/medsci12030032

**Published:** 2024-07-02

**Authors:** Evgeniy Nikolaevich Goncharov, Oleg Aleksandrovich Koval, Eduard Nikolaevich Bezuglov, Aleksandr Aleksandrovich Vetoshkin, Nikolay Gavriilovich Goncharov, Manuel De Jesus Encarnación Ramirez, Nicola Montemurro

**Affiliations:** 1 Russian Scientific Center of Surgery Named after Academician B. V. Petrovsky, 121359 Moscow, Russia; 2 Department of Sports Medicine and Medical Rehabilitation, Sechenov First Moscow State Medical University, 119435 Moscow, Russia; 3 The Nikiforov Russian Center of Emergency and Radiation Medicine, 187015 St. Petersburg, Russia; 4 Russian Medical Academy of Continuous Professional Education, 119435 Moscow, Russia; 5 Department of Neurosurgery, RUDN University, 121359 Moscow, Russia; 6 Department of Neurosurgery, Azienda Ospedaliero Universitaria Pisana (AOUP), 56100 Pisa, Italy

**Keywords:** avascular necrosis, femoral head, anatomy, conservative treatment, surgery

## Abstract

Introduction: Avascular necrosis (AVN) of the femoral head is a pressing orthopedic issue, leading to bone tissue death due to disrupted blood supply and affecting the quality of life of individuals significantly. This review focuses on conservative treatments, evaluating their efficacy as mainstay therapies. Enhanced understanding of AVN’s pathophysiology and advancements in diagnostic tools have rekindled interest in non-surgical interventions, emphasizing personalized, multidisciplinary approaches for improved outcomes. Material and Method: A systematic search was conducted on PubMed, SCOPUS, and Google Scholar databases from January 2020 to August 2023, with the objective of focusing on conservative treatments for AVN of the femoral head. Eligible studies, including original research, case reports, and observational studies, were examined for relevant, well-documented patient outcomes post-conservative treatments, excluding non-English and surgically focused articles without comparative conservative data. Results: A systematic search yielded 376 records on AVN of the femoral head across multiple databases. After de-duplication and rigorous screening for relevance and quality, 11 full-text articles were ultimately included for a comprehensive qualitative synthesis, focusing on conservatively managing the condition. Conclusions: This review evaluates the effectiveness of conservative treatments such as pharmacological interventions and physical modalities in managing AVN of the femoral head. Despite promising results in symptom alleviation and disease progression delay, variability in outcomes and methodological limitations in studies necessitate further rigorous, randomized controlled trials for a robust, patient-centric approach to optimize therapeutic outcomes in AVN management.

## 1. Introduction

Avascular necrosis (AVN) of the femoral head, colloquially known as osteonecrosis, represents a significant orthopedic challenge characterized by the death of bone tissue resulting from a disruption in blood supply [1]. Such an ischemic event often culminates in debilitating pain, joint dysfunction, and, in later stages, joint collapse, substantially impacting the affected individual’s quality of life [2]. While several etiological factors—encompassing trauma, corticosteroid use, excessive alcohol consumption, and various systemic conditions—have been linked to AVN [3], the overarching concern for clinicians remains its effective management.

Traditionally, the spectrum of management for AVN has ranged from surgical interventions, such as core decompression and total hip arthroplasty, to conservative treatments. The latter, which includes modalities like weight-bearing restrictions, pharmacotherapy, and physical therapy, has primarily been employed in the early stages of the disease [4]. As the prevalence of AVN continues to rise, especially in younger populations, the emphasis on understanding and optimizing conservative treatments becomes paramount [5].

Conservative treatments, historically seen as interim solutions or suited for early-stage AVN, are now being rigorously evaluated for their potential as mainstay therapies. This resurgence in interest is primarily due to advancements in understanding the disease’s pathophysiology and the realization that early interventions can significantly alter its course [6]. For instance, there is growing evidence that judiciously managed non-surgical interventions can not only alleviate symptoms but may also prevent or delay the need for more invasive procedures, particularly in younger patients who may be looking at several decades of joint usage [7]. Furthermore, the patient-centered approach in modern healthcare underscores the need for interventions that are not only effective but also align with the patients’ lifestyles, occupational needs, and long-term health goals [8]. Conservative treatments often meet these criteria, as they tend to be less invasive, have reduced recovery times, and align more seamlessly with patients’ daily lives compared to surgical options. The burgeoning field of regenerative medicine also raises the possibility of utilizing the body’s own healing mechanisms, a prospect that remains deeply intertwined with conservative strategies [9].

Yet, despite their apparent advantages, conservative treatments are not without their challenges. Diverse patient presentations, varying stages of the disease at the time of diagnosis, and the multifactorial nature of AVN make it imperative that these treatments are personalized, optimized, and continually assessed against emerging evidence [10]. It is within this context that this review positions itself, endeavoring to map the landscape of conservative treatments in AVN of the femoral head. Indeed, as conservative treatments gain traction in managing AVN, a multidisciplinary approach is becoming increasingly essential. Rheumatologists, orthopedic surgeons, and even nutritionists are now often collaborating in crafting holistic treatment regimens. This teamwork reflects the understanding that AVN is not merely an orthopedic concern but has metabolic, genetic, and lifestyle elements that require comprehensive management [11].

Another driving factor is the evolution of diagnostic tools. Enhanced imaging modalities like MRI and advanced biochemical markers can now identify AVN at even subtler stages, offering a wider window for conservative treatments to exert their benefits [12]. The intersection of technology and therapeutics promises not only improved outcomes but also the potential for predictive and preventive strategies.

The socio-economic aspect of AVN cannot be ignored. With surgical interventions often being cost-intensive, the pursuit of effective conservative strategies becomes crucial from a healthcare economics perspective [13]. In many parts of the world, where access to advanced surgical facilities might be limited or cost-prohibitive, well-structured conservative treatments can be life-altering, offering pain relief and functional restoration [14].

Lastly, patient empowerment and education play an indispensable role. In an era where patients are increasingly involved in shared decision-making, understanding the spectrum, efficacy, and limitations of conservative treatments is vital. This not only facilitates informed choices but also aligns expectations, promotes adherence, and optimizes outcomes [2].

The objective of this comprehensive literature review is to compare and contrast the efficacy, limitations, and evolving evidence of conservative treatments for AVN. Through this, we aim to equip clinicians and researchers with a consolidated knowledge framework to inform treatment choices and guide future research directions.

## 2. Materials and Methods

### 2.1. Search Strategy

A comprehensive and systematic search of the following electronic databases was conducted: PubMed, SCOPUS, and Google Scholar, covering the literature from January 2020 to August 2023. The search was structured using combinations of the following terms and their synonyms: “avascular necrosis” OR “osteonecrosis”, “femur” OR “femoral head”, “conservative treatment” OR “non-operative management”.

### 2.2. Inclusion and Exclusion Criteria

The inclusion criteria were original research articles, case reports, cohort studies, and observational studies that clearly documented patient outcomes after conservative treatments for AVN of the femoral head. The exclusion criteria were articles not written in English, studies focused exclusively on surgical interventions for AVN without a comparative conservative group, studies with a lack of relevance to the AVN of the femoral head, and letters to the editor.

### 2.3. Data Extraction

Two independent reviewers (E.G. and R.N.) meticulously extracted the following data from eligible studies: authors, publication year, study design, patients, details of conservative interventions employed, outcomes, follow-up, and adverse events. Inconsistencies between reviewers were settled through mutual discussion and, if necessary, mediation by a third reviewer (N.M.).

## 3. Results

**Figure 1:**
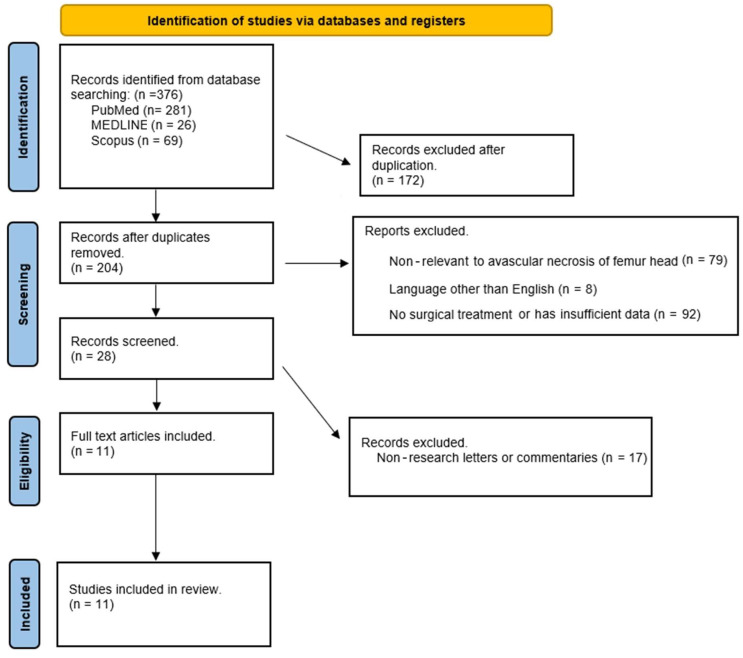
PRISMA flow diagram of the articles screened.

The systematic search strategy resulted in the identification of a total of 376 records across multiple databases, which included PubMed (n = 281), MEDLINE (n = 26), and Scopus (n = 69). In the subsequent de-duplication process, 172 duplicate records were identified and removed, leaving 204 unique records eligible for screening. The remaining records underwent a rigorous screening process based on their relevance to the AVN of the femoral head. A total of 179 records were excluded during this phase due to various reasons, such as: lack of relevance to the AVN of the femoral head (n = 79); publication language other than English (n = 8); and insufficient data or focus on non-surgical treatments (n = 92). This led to the inclusion of 28 records, which were further subjected to an eligibility assessment. During this assessment, 17 records, primarily consisting of non-research letters or commentaries, were excluded from the review. Consequently, a total of 11 full-text articles were deemed eligible and included in the final qualitative synthesis, ensuring that the review was comprehensive and based on relevant and sufficient data pertaining to the AVN of the femoral head (Figure 1) [2,14,15,16,17,18,19,20,21,22,23]. Table 1 shows all the details of the included studies.

## 4. Discussion

This comprehensive literature review delineated the potential of conservative treatments in managing AVN of the femoral head. The discussed studies exhibit a wide array of conservative interventions, underscoring their potential for symptom alleviation and decelerating disease progression, especially in the early stages. The heterogeneity in treatment outcomes, largely attributed to the disease stage at intervention onset, emphasizes the necessity for personalized treatment regimens. The multidisciplinary approach, emerging diagnostic tools, and socio-economic considerations further highlight the complexity and the requisite comprehensive approach to managing AVN. The promising avenues of regenerative medicine and patient-centric approaches denote a paradigm shift towards more sustainable and less invasive management strategies, fostering a collaborative effort to mitigate the orthopedic and systemic ramifications of AVN.

### 4.1. Pathophysiology of AVN

Bone tissue necrosis follows a similar pattern in both adults and children, yet notable differences exist due to the varying levels of cartilage maturity in the femoral head. In children, the epiphysis and proximal femoral physis remain active, potentially increasing the likelihood of bone regeneration compared to adults, whose bone growth is complete. The pathophysiology of this condition is not fully understood, but it typically unfolds in two main phases. Initially, there is an ischemic phase where the blood supply to the bone is compromised, followed by a potential regeneration phase where the affected bone may begin to heal [24,25,26].

#### 4.1.1. Ischemia

The onset of ischemia in AVN often goes unrecognized until symptoms develop, making it difficult to pinpoint its exact start. Etiology, particularly in pediatric non-traumatic AVN, remains elusive, with several theories such as vascular disruption, thrombosis, and direct cartilage damage being considered [26,27]. A constitutional theory posits that abnormal cartilage growth can destroy the blood supply to the epiphysis, leading to ischemia in the femoral head [15]. Diagnosis is often sought after the disease has progressed beyond the regeneration phase, indicating that initial ischemia may be symptomatically minimal.

**Figure 2:**
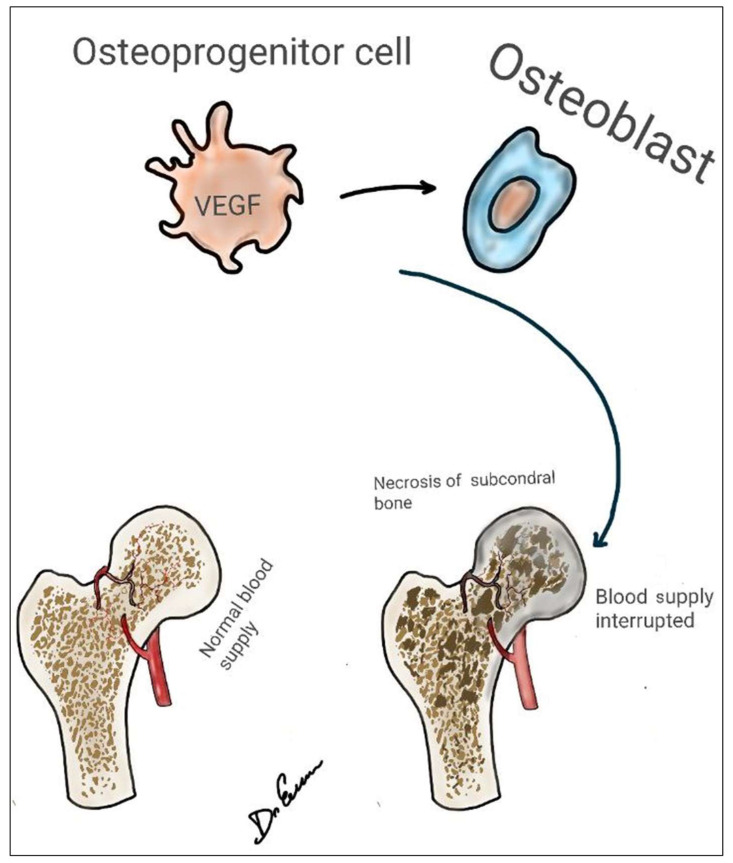
Avascular necrosis of femoral head pathophysiology. After ischemia, hypoxic vascular endothelial growth factor (VEGF).

Diagnostic tools like X-ray imaging lack sensitivity to early ischemic changes as they do not affect the mineral content of the bone [28]. More sensitive techniques like bone scintigraphy and MRI are preferred for early detection, showing decreased blood flow and changes in bone marrow, respectively [29]. A definitive diagnosis is confirmed through histology, which can reveal necrotic cellular changes and the absence of viable osteocytes within bone lacunae [30]. Studies also note the impact of inherited thrombosis on venous occlusion and subsequent necrosis [27], with a significant decrease in endothelial progenitor cells contributing to failed neo-angiogenesis in AVN progression (Figure 2) [31].

#### 4.1.2. Regeneration

After the blood supply to the femoral head is disrupted, initiating necrosis, molecular signals recruit mesenchymal stem cells (MSCs) to the necrotic site, influenced by cartilage-piercing blood vessels aligned with the medial circumflex artery [32]. This mechanism is vital for therapeutic approaches [33]. Previous studies indicated that premature revascularization and abnormal cartilage growth could enlarge the femoral head, increasing its vulnerability [32]. This excessive neovascularization introduces MSCs and monocytes that aid in bone remodeling, where two simultaneous processes occur: osteoclasts derived from monocytes resorb the outer subchondral bone, while osteoblasts build tissue at the core [9]. This leads to subchondral bone degeneration, observable as a subchondral fracture line on X-rays [28], causing collapse of the overlying articular cartilage. Although cartilage is not directly affected by AVN, it suffers from the collapse of the supportive subchondral bone [33]. Successful recovery of the femoral head’s shape and height—biological plasticity—requires containment treatment to ensure full acetabular coverage and joint mobility, often achieved through surgical interventions. Additionally, while scintigraphy can detect revascularization in both necrotic and pre-necrotic stages, making early AVN diagnosis challenging, it, along with MRI, remains critical for identifying these changes before significant bone and cartilage damage ensues [34,35].

#### 4.1.3. Cell and Tissue Necrosis

AVN follows a complex pattern of cellular death and subsequent bone tissue formation and resorption [36]. The process begins with the necrosis of adipocytes and hematopoietic cells, quickly followed by interstitial marrow edema. Osteocytes begin to die within 2–3 h of oxygen depletion, resulting from blood supply disruption, although histological signs like nuclear pyknosis and empty bone lacunae become evident only after 24–72 h [37]. Nuclear pyknosis leads to the irreversible condensation of chromatin, followed by nuclear fragmentation. Concurrently, cellular organelles swell, rupture, and are eventually cleared by phagocytosis. Subsequent repair processes involve capillary revascularization and reactive hyperemia around the necrotic areas, initiating both bone resorption and new bone formation to remodel the dead tissue. New bone overlays the dead trabeculae, with only partial resorption occurring. However, the destruction of subchondral bone primarily results from an imbalance between bone resorption and formation, leading to weakened bone trabeculae, subchondral fractures, and joint collapse [38]. In silico studies using finite element modeling have shown that subchondral fractures result from reduced integrity of the cancellous subchondral bone trabeculae compared to the subchondral plate [39]. X-ray imaging reveals these changes as areas of lucency indicating increased bone resorption and areas of sclerosis showing trabeculae either dead or undergoing repair [40].

### 4.2. Management

#### 4.2.1. Non-Surgical Management

Gómez et al. (2013) emphasized that the primary objective of non-surgical or conservative treatment approaches, such as restricting weight-bearing, is to enhance hip function, mitigate pain, and postpone the femoral head’s collapse and necrotic alterations. This is particularly relevant in the initial phases of AVN when patients do not have a history of trauma [41]. Restriction in weight-bearing using a cane, crutches, or walker is one of the ways to delay disease progression. However, some papers have indicated that reducing joint reactive forces does not slow disease progression [42].

#### 4.2.2. Pharmacological Treatment

A variety of pharmacological interventions, including the utilization of bisphosphonates, statins, and vasodilators, among others, have been dissected by Sen et al. (2009). They discuss how these treatments are employed in the early stages of AVN, but their effectiveness remains encumbered by limited evidence and a lack of explicit guidelines, eventually leading many patients towards surgical interventions [43,44]. Several pharmaceutical agents and biological therapies have been employed across different studies. For example, Fang et al. [15] and Konarski [2] focused on using celecoxib, anticoagulants, and other agents, reflecting a focus on pharmacologically manipulating biological pathways involved in AVN, such as inflammation and coagulation. Shankar et al. [20] and Yang et al. [21] emphasized the use of cellular therapies such as the transplantation of differentiated osteoblasts and bone marrow-derived stromal stem cells (BMSCs), signifying a shift towards leveraging the regenerative potential of cellular components in mitigating the effects of AVN.

#### 4.2.3. Bisphosphonates

Bisphosphonates are recommended in the early stages of AVN. They act by inhibiting osteoclastic activity and reducing bone turnover, thus preventing woven bone formation [29]. In a randomized controlled trial, the efficacy of alendronate and placebo was compared in patients with non-traumatic AVN at Steinberg stages II–III. Patients in the drug arm experienced two collapses out of 29 assessed femoral heads, while 19/25 assessed femoral heads collapsed in the placebo arm [45]. However, another prospective, randomized, placebo-controlled study by Chen et al. did not support these findings. There were no significant differences in radiographic outcomes, prevention of THA, or improvement of quality of life between the placebo and treatment arms [46,47]. The results of the available studies are therefore inconclusive. Some of them have limitations in their methodology, including the lack of a control group. The paucity of available evidence does not allow for the formation of guidelines for the dose and duration of bisphosphonate therapy.

#### 4.2.4. Statins and Vasodilators

Therapy with statins may inhibit corticosteroid-induced adipogenesis and osteonecrosis of the femoral head. Nonetheless, similarly to bisphosphonate therapy, there are no guidelines on statin use. The results of Ajmal et al. indicated no difference in the occurrence of osteonecrosis between patients on corticosteroids receiving or not receiving statins [48]. In contrast, Prichett et al. [49] observed a significant reduction in the AVN rate in patients on steroids and receiving statins. The beneficial effect of a vasodilator iloprost on radiographic and clinical outcomes in patients with early stages of AVN was reported. Claßen et al. investigated the effect of iloprost in 108 patients with osteonecrosis; the median follow-up of patients was 49.7 months. Most of the patients (74.8%) noted an improvement in subjective complaints and a decrease evaluated by the visual analog scale. However, patients with a lower stage of disease had better outcomes [50]. Some authors suggest that enoxaparin may delay the progression of osteonecrosis if therapy is implemented in the early stages of the disease [51], but data on its effectiveness remain limited.

#### 4.2.5. Other Therapies

Different shockwave devices were studied in AVN treatment. Several studies involving extracorporeal shockwave therapy (ESWT) in AVN with promising results have been published [52]. The main effect observed was a decrease in pain; some patients had a complete regression of MRI changes. ESWT’s proposed mechanism of action is the stimulation of osteoblastic activity, which results in increased bone density in the pelvic area. Russo et al. stated that ESWT efficacy is more significant in the early stages of the disease and that ESWT is more effective than core decompression and grafting [53]. A substantial focus was also given to ESWT in the studies by Wang et al. [16] and Chen et al. [17]. ESWT appears to be a prominent non-invasive modality, with varying levels of efficacy reported. It has been highlighted that the therapeutic benefits of ESWT might be influenced by factors such as the stage of the disease, with earlier stages responding more favorably.

Alves et al. [54] and Zhang et al. [55] investigated shock wave therapy for treating AVN of the femoral head, utilizing its high-pressure acoustic waves to promote osteogenesis and neovascularization. Alves et al.’s review [54] assessed five studies, finding shock wave therapy superior to core decompression and alendronate in improving functional and radiological outcomes. Most patients experienced positive effects within 2–3 years. Zhang et al.’s broader review [55] encompassed 17 studies comparing the effectiveness of extracorporeal shock wave therapy (ESWT) alone or combined with surgical interventions such as core decompression and multiple drilling, with or without bone grafting. They concluded that shock wave therapy not only provided more benefits than surgical options but also enhanced results when combined with pharmacological treatments. This method is suggested as a highly effective modality for AVN management, demonstrating significant long-term benefits.

#### 4.2.6. Hyperbaric Oxygen Therapy

HBO therapy, as explored by Moghamis et al. [22] and Salameh et al. [23], emerges as an alternative non-invasive therapeutic option, demonstrating safety and satisfactory outcomes and emphasizing its potential role in the pre-collapse stages of AVN. Li et al. [56] and Paderno et al. [57] explored the efficacy of hyperbaric oxygen therapy (HBOT) for treating all stages of femoral head osteonecrosis through their systematic reviews. Li et al. [56] concluded that HBOT led to significant clinical improvements compared to controls by enhancing tissue oxygen partial pressures and stimulating osteoblasts, osteoclasts, fibroblasts, and bone morphogenic proteins. This resulted in neo-vasculogenesis, osteogenesis, reduced inflammation, inflammatory markers like TNF and IL-6, and increased bone formation markers. Paderno et al. [57] confirmed these findings, noting statistically significant functional improvements in HBOT patients. They proposed a protocol of 60–90 daily HBOT sessions at 2–2.5 atmospheres for an hour each, though the treatment costs between $6000 and $9000 and requires specialized equipment. HBOT is also recognized for treating conditions such as carbon monoxide poisoning, radio necrosis, gas embolism, decompression sickness, and burns. Its application in femoral head osteonecrosis, as endorsed by the tenth European consensus conference on hyperbaric medicine [49], is as an adjunct therapy, broadening its use in clinical settings. Recent studies in tissue engineering and regenerative therapies are advancing our understanding of AVN treatments. These efforts focus on refining the properties of engineered materials to enhance regenerative capabilities, bolstered by a deepening grasp of the disease’s pathobiology [58,59]. Key technologies employed include cellular therapies using bone marrow-derived mesenchymal stem cells (BMSCs), growth factor therapies, metallic implants, and advanced manufacturing techniques such as 3D bioprinting and nonoprinting for crafting ceramic and polymeric scaffolds. While these technologies remain experimental, their potential advantages and disadvantages are actively being explored to optimize regenerative outcomes for AVN [60,61].

#### 4.2.7. Cellular Therapies

Cellular therapies are particularly effective for early-stage AVN, especially stage 2 [62]. These therapies utilize mesenchymal stem cells (MSCs), which are crucial for the regeneration of bone and cartilage. MSCs are typically harvested from bone marrow through aspirates, cultures, or concentrates and can also be derived from adipose tissue or the umbilical cord [63].

In addition to MSCs, adipose-derived stem cells (ADSCs) are explored for their regenerative potential in AVN. These cells can be administered intra-arterially or directly into the necrotic zone to leverage their multipotentiality and paracrine signaling abilities, which enable them to target and repair injured tissues [64]. MSCs are noted for their role in bone regeneration and initiating the revascularization of necrotic tissues in AVN. They regulate both bone formation and resorption by secreting various cytokines such as IL-1β, IL-6, IL-11, and osteoprotegerin (OPG); growth factors like PDGF, TGF-β, and FGF-2; and chemokines including RANKL, which are instrumental in these processes [65,66]. These cells also influence osteoclast activity through the NF-κB signaling pathway, where the receptor activator of nuclear factor kappa-Β (RANK) promotes and OPG inhibits osteoclast formation [65]. The use of cellular therapies offers a less invasive alternative to surgical interventions, highlighting their potential as a transformative approach to treating AVN.

Pak et al. presented two case reports investigating the regenerative potential of adipose tissue-derived stem cells combined with platelet-rich plasma for bone healing. In these studies, the researchers observed the formation of medullary bone-like tissue within the necrotic regions of the femoral head in both patients, demonstrating promising results in bone regeneration [67,68].

Recently, genetic engineering techniques have been applied to enhance the capabilities of mesenchymal stem cells (MSCs) for bone regeneration, particularly in the context of femoral head necrosis. By genetically modifying MSCs to overexpress key growth factors like vascular endothelial growth factor (VEGF), fibroblast growth factor (FGF), and bone morphogenetic protein (BMP), researchers have significantly improved the regenerative abilities of these cells. The overexpression of these factors boosts cellular signaling, attracting more cells to the damaged area and enhancing anabolic activities, including bone formation and vascularization. Evidence of this approach’s effectiveness was demonstrated in a rabbit model, where MSCs transfected with FGF-2 and implanted in a xenogeneic antigen-cancellous bone (XACB) scaffold showed enhanced bone regeneration. The increase in FGF-2 expression was observed to suppress TNF-α, a pro-inflammatory cytokine, thereby improving bone regeneration in a model of steroid-induced osteonecrosis [69,70]. Another study utilized MSCs from bone that were genetically engineered to express both VEGF and BMP-6 and combined these with a polylactide-co-glycolide (PLAGA) hydrogel. When implanted subcutaneously in nude mice, this combination led to notable increases in bone formation and angiogenesis after four weeks, underscoring the therapeutic potential for AVN treatment [71,72]. Further research involved adenovirus-mediated expression of BMP-2 and basic FGF in bone marrow stem cells (BMSCs) used in conjunction with demineralized bone matrix (DBM) in a canine model. This innovative approach resulted in significantly increased bone regeneration, enhanced vascularization, and improved mechanical properties of the bone, such as bending and compressive strength, compared to controls in the AVN model [73].

MSCs have some disadvantages linked to their low yield and painful extraction process, which can involve surgical complications. ADSCs, which can be easily isolated and have a significantly greater yield than MSCs, have thus been explored for the regeneration of bone in AVN. It has been demonstrated that osteogenically induced ADSCs can induce bone regeneration in a rabbit model [74]. A clinical study demonstrated the use of ADSCs in two patients, where autologous ADSCs were injected into the affected hips and the patients were examined after 3 months. Other stem cells, such as dental-pulp stem cells (DPSCs), synovial-derived mesenchymal stem cells (SDMSCs), blood-derived mesenchymal stem cells (BDMSCs), and umbilical cord-derived mesenchymal stem cells (UCSMSCs), have also been explored for bone regeneration in AVN [75].

### 4.3. Growth Factor Therapies

Growth factors play a crucial role in enhancing stem cell differentiation and vasculogenesis, which is vital for osteogenesis and bone healing. Key growth factors such as bone morphogenetic protein (BMP), vascular endothelial growth factor (VEGF), hepatocyte growth factor (HGF), and platelet-derived growth factor effectively stimulate mesenchymal stem cells (MSCs) to differentiate into osteoblasts and chondroblasts [76]. These factors are particularly advantageous in treating the AVN of the femoral head, as they can be administered non-invasively through injections or used in conjunction with surgical treatments and tissue-engineered grafts or scaffolds. This method avoids the need for additional surgical interventions, simplifies treatment, and potentially enhances recovery outcomes. The use of growth factors for the regeneration of the bone and vasculature of the necrotic femoral heads has been practiced clinically. They can be injected or delivered through overexpression by genetically transfected stem cells (Table 2) [76].

Bone morphogenetic proteins (BMPs) play a crucial role in stimulating mesenchymal progenitor cells to form bone and cartilage, offering significant benefits in conditions like AVN of the femoral head [89,90]. Specifically, BMP subsets 2, 6, and 7 have shown high efficacy in this regard [75]. These BMPs are often used alongside vascular endothelial growth factor (VEGF), an angiogenic factor that aids in vascularization [76,77]. A study by Ma et al. involving 36 rabbits with induced AVN demonstrated that combining BMP-2 and VEGF-165 with bone marrow stem cells (BMSCs) during core decompression resulted in enhanced bone repair and vasculogenesis compared to other methods, highlighting the potent synergistic effects of BMP and VEGF in promoting differentiation and angiogenesis [91].

Additionally, hepatocyte growth factor (HGF), another endothelial growth factor, has been identified as a strong promoter of vasculogenesis and cell differentiation, potentially more so than VEGF [76,77]. High concentrations of HGF were particularly effective in osteogenic differentiation of MSCs and tissue repair in rabbit models [88]. Wilczyński and Kasprzak [92] evaluated the dynamics of isometric changes in strength and muscular lumbar–pelvic imbalances in the treatment of women with low back pain. In a related study by Wen et al. [93], combining HGF with fibrin glue—a supportive material for cell differentiation—significantly enhanced cell differentiation and vasculogenesis in MSCs derived from rabbits. This combination was evaluated in 30 rabbit models and was found to effectively support the differentiation and regeneration of femoral head necrosis [93]. These studies collectively underscore the potential of growth factors like BMP, VEGF, and HGF in tissue engineering applications, particularly in the treatment and regeneration of bone and cartilage tissues [94,95].

### 4.4. Limitations of the Study

Heterogeneity of the Included Studies: There is a marked heterogeneity in the types of studies included in this review, ranging from case reports and experimental studies to prospective studies and reviews. The diversity in study designs can make it challenging to draw comprehensive conclusions or comparisons.

Inclusion of Animal and In Vitro Studies: This review includes studies involving animals and in vitro models, which may not directly translate to human physiology and pathology, limiting the applicability of findings to human patients.

Varied Interventions and Outcomes: The interventions in the reviewed studies are varied, ranging from pharmacological treatments like bisphosphonates and statins to physical modalities like extracorporeal shockwave therapy (ESWT) and hyperbaric oxygen therapy (HBO). This vast range of treatments can make it difficult to delineate which conservative treatment modalities are most effective.

Lack of Randomized Controlled Trials (RCTs): This review seems to have a limited number of randomized controlled trials, which are crucial for establishing the efficacy of therapeutic interventions. The absence of RCTs may affect the strength of the recommendations and conclusions drawn.

## 5. Conclusions

This comprehensive literature review aimed to elucidate the efficacy and applicability of conservative treatments in managing AVN of the femoral head. Throughout various studies, it was underscored that conservative approaches, ranging from pharmacological interventions, such as bisphosphonates and statins, to physical modalities like extracorporeal shockwave therapy (ESWT) and hyperbaric oxygen therapy (HBO), hold substantial promise, particularly in the early stages of the disease. The conservative treatments were primarily geared towards symptom alleviation, delaying the progression of the disease, and enhancing the overall quality of life of the affected individuals. The results elucidate a nuanced landscape of conservative management strategies, marked by a pronounced heterogeneity in treatment outcomes. This variability is notably influenced by the disease stage at the initiation of the intervention and the specific therapeutic modalities employed. Several studies have heralded the potential of regenerative and cellular therapies, highlighting the emergence of innovative paradigms in the conservative management of AVN. However, it is imperative to underscore the presence of notable limitations within the available literature, including a predominance of studies with smaller sample sizes and varying degrees of methodological rigor. The existence of such constraints necessitates a cautious interpretation of the findings and calls for further well-designed, randomized controlled trials to bolster the evidence base supporting conservative treatment strategies in AVN of the femoral head. Future research should focus on standardizing treatment protocols and exploring the full potential of regenerative therapies.

## Figures and Tables

**Table 1:** Studies included in the review.

Author [Ref.] (Year)	Study Design	Participant	Details of Conservative Interventions Employed	Outcomes Measures	Duration of Follow-Up	Reported Adverse
Wang et al. [14] (2014)	Review	160 hips	ACTH and VEGF	Conservative treatment may be a major focus for orthopedic studies in the future. The principle of the treatment is to provide mechanical support to prevent collapse of the femoral head, improve the speed and quality of repair at the molecular level, increase osteoclast apoptosis, and reduce osteoblast and osteocyte apoptosis.	1–3 years	No adverse event
Konarski [2] (2022)	Review	46 hips	Anticoagulants, statins, vasodilators, bisphosphonates	Non-operative management should be performed in patients with early-stage disease, while surgical treatment is routinely used in more advanced stages.	1–2 years	No adverse event
Fang et al. [15] (2020)	Prospective study	30 participants, 41 hips	Celecoxib, salvia miltiorrhiza, tetramethylypyrazine, and a reduction in weight-bearing activities	Final follow-up rates of femoral head survivorship were 4.9% in the non-surgical group and 36.7% in the surgical group. The Harris hip score was significantly improved following surgery when compared with non-surgical treatment (*p* < 0.05). The results indicated that core decompression and porous tantalum rod implantation are beneficial short- and mid-term treatment methods for AVN of the femoral head.	18 months	No complications, including infection, delayed healing, or fractures, were reported.
Wang et al. [16] (2008)	Prospective	48 patients, 60 hips	All patients were treated with 6000 impulses of ESWT at 28 kV (equivalent to 0.62 mj/mm^2^) to the affected hip in a single session. Patients in group B also received alendronate 70 mg per week for 1 year, whereas patients in group A did not.	ESWT and alendronate produced comparable results as compared with ESWT without alendronate in early ONFH. ESWT is effective with or without the concurrent use of alendronate. The joint effects of alendronate over ESWT in early ONFH are not realized in the short term.	1 year	No adverse event
Chen et al. [17] (2009)	Prospective	17 patients with bilateral hip necrosis	On the ESWT side, each hip received 6000 impulses of shockwave at 28 kV.	The evaluations included a pain score, a Harris hip score, radiographs, and MR images. The magnitudes of improvement in pain and function favored the ESWT side. Thirteen patients rated ESWT better than THA; four patients reported comparable results between THA and ESWT; and none graded THA better than ESWT. Better functional outcomes were observed after ESWT for early hip necrosis than THA for late cases in patients with bilateral hip disease.	6 months	No adverse event
Kusz et al. [18] (2012)	Prospective	18 patients	Each spot received a dose of 1500 pulses at an energy flux density of 0.4 mj/mm^2^ and a frequency of 4 Hz. Each patient underwent 5 therapy sessions.	Extracorporeal focused shockwave therapy resulted in considerable improvement in the patients’ quality of life at 6 weeks’ follow-up. At 6 months, some patients reported intensified pain and worse hip function.	12 months	Pain and worse hip function
Lebouvier et al. [19] (2015)	Prospective	10 pigs	Injection of osteoprogenitor cells like BMSCc	Intra-osseous injection of BMSCs in FH seems to be a good strategy for ONFH treatment, as the safety of the biodistribution of BMSCs is ensured. Moreover, the efficacy of BMSCs in natural ONFH seems to indicate that this is a promising approach. Altogether, these results constitute the preclinical data necessary for the setup of a clinical application with expanded BMSCs in the context of advanced therapeutic medicinal products.	9 weeks	No adverse event
Shankar et al. [20] (2023)	Case report	44-year-old	Aalcos	Biological therapy with differentiated osteoblasts remains a viable option for AVN of the femoral head when compared with an undifferentiated BMAC cocktail.	6 years	No adverse event
Yang et al. [21] (2018)	Experimental research	25 rats	BMSCs, GFP, stromal-cell-derived factor (SDF)-1	SDF-1α overexpression in BMSCs promotes bone generation as indicated by osteogenesis and angiogenesis, suggesting SDF-1α may serve as a therapeutic drug target for ONFH treatment.	6 weeks	No adverse event
Moghamis et al. [22] (2021)	Retrospectively	19 patients	HBO	Hyperbaric oxygen therapy could be used as an alternative, non-invasive treatment option.	12 months	No adverse event
Salameh et al. [23] (2021)	Case report	15 patients	HBO	Hyperbaric oxygen treatment for pre-collapse AVN of the femoral head is considered a safe alternative with satisfactory clinical and radiological outcomes and a low complication rate.	22 months	No complications were reported in all patients.

BMSCs, bone marrow mesenchymal stromal cells; HBO, hyperbaric oxygen therapy; GFP, green fluorescent protein; ESWT, extracorporeal shockwave therapy; ACTH, adrenocorticotropic hormone; VEGF, vascular endothelial growth factor.

**Table 2:** Growth factor therapies.

Growth Factor	Associated Cells	Delivery Strategy	Regeneration Results
Hepatocyte growth factor (HGF) [76]	BMSCs	HGF transgenic BMSCs transplanted using core decompression (CD) with fibrinogen drug delivery mixture (FG)	Formation of new capillaries on the bone plates of the trabeculae. Bone marrow is rich in hematopoietic tissue.
Granulocyte colony stimulating factor (G-CSF) and stem cell factor (SCF) [77]		G-CSF and SCF injected subcutaneously for 5 days, mobilizing BMSCs	Increase in osteocalcin protein expression. Vessel formation was 3.3 fold greater, and vessel density was 2.6 fold greater than the control.
Vascular endothelial growth factor (VEGF) [78]		Plasmid encoding VEGF immobilized on a cartilage carrier into the necrotic area of the femoral head	Increase in bone formation after 8 weeks.
Bone morphogenetic protein (BMP-2) [79]	BMSCs	Modified BMSCs loaded onto the *β*-TCP cylinder and implanted into the core tract from CD	Increased amounts of new bone and higher maximum compressive strength and bone density.
BMP-2 and BMP-14 [80]		BMP-laden collagen scaffolds transplanted following CD	BMP-14-loaded scaffolds improved bony remodeling of the necrotic area.
VEGF [81]		VEGF injected continuously or through an osmotic micropump	Reversal of osteonecrosis.
Recombinant human fibroblast growth factor (rhFGF)-2 [82]		rhFFGF-2-impregnated gelatin hydrogel administered locally	Increased Harris hip score. Reduction in pain level.
VEGF [83]		Deproteinized bone (DPB) with the recombinant plasmid pcDNA3.1-hVEGF165 was implanted into the drilled tunnel of the necrotic femoral head	Increased bone formation and capillary vessel regeneration.
VEGF [84]	BMSCs	Transgenic autologous BMSCs implanted following CD	Enhanced bone reconstruction and blood vessel regeneration.
rhBMP-2 [85]		Cavity was made using the light bulb technique, and an autologous cancellous bone combination of rhBMP-2 filled the cavity	May be effective in avoiding future THR in younger patients and improving the speed of bone repair (lack of statistical significance).
rhBMP-7 [86]		Fibular graft harvested from the femoral neck, sprinkled with rhBMP-7, and implanted in the tunnel	Increased Harris hip score. Decrease in pain. Retention in the sphericity of the femoral head.
BMP-2 [87]		Percutaneous intraosseous injection of BMP-2 and ibandronate	Decreased femoral head deformity and increased bone formation.
HGF [88]	MSCs	Transplantation of HGF-transgenic MSCs through the CD tunnel	Increased the number of MSCs and osteogenic differentiation of MSCs.

## Data Availability

Data are contained within the article.
